# Application of Modeling Approaches to Explore Vaccine Adjuvant Mode-of-Action

**DOI:** 10.3389/fimmu.2019.02150

**Published:** 2019-09-12

**Authors:** Paul R. Buckley, Kieran Alden, Margherita Coccia, Aurélie Chalon, Catherine Collignon, Stéphane T. Temmerman, Arnaud M. Didierlaurent, Robbert van der Most, Jon Timmis, Claus A. Andersen, Mark C. Coles

**Affiliations:** ^1^Kennedy Institute of Rheumatology, University of Oxford, Oxford, United Kingdom; ^2^Department of Electronic Engineering, University of York, York, United Kingdom; ^3^GSK, Rixensart, Belgium; ^4^Faculty of Technology, University of Sunderland, Sunderlandm, United Kingdom

**Keywords:** vaccines, adjuvants, mathematical modeling, computational biology, systems biology, mechanistic modeling, AS01

## Abstract

Novel adjuvant technologies have a key role in the development of next-generation vaccines, due to their capacity to modulate the duration, strength and quality of the immune response. The AS01 adjuvant is used in the malaria vaccine RTS,S/AS01 and in the licensed herpes-zoster vaccine (Shingrix) where the vaccine has proven its ability to generate protective responses with both robust humoral and T-cell responses. For many years, animal models have provided insights into adjuvant mode-of-action (MoA), generally through investigating individual genes or proteins. Furthermore, modeling and simulation techniques can be utilized to integrate a variety of different data types; ranging from serum biomarkers to large scale “omics” datasets. In this perspective we present a framework to create a holistic integration of pre-clinical datasets and immunological literature in order to develop an evidence-based hypothesis of AS01 adjuvant MoA, creating a unified view of multiple experiments. Furthermore, we highlight how holistic systems-knowledge can serve as a basis for the construction of models and simulations supporting exploration of key questions surrounding adjuvant MoA. Using the Systems-Biology-Graphical-Notation, a tool for graphical representation of biological processes, we have captured high-level cellular behaviors and interactions, and cytokine dynamics during the early immune response, which are substantiated by a series of diagrams detailing cellular dynamics. Through explicitly describing AS01 MoA we have built a consensus of understanding across multiple experiments, and so we present a framework to integrate modeling approaches into exploring adjuvant MoA, in order to guide experimental design, interpret results and inform rational design of vaccines.

## Introduction

Adjuvants are immunostimulants that shape and enhance the immune response to antigens through mimicking key aspects of innate pathogen recognition, leading to robust long-term memory recall responses (1, 2). Many modern vaccine adjuvants activate pattern-recognition-receptors (PRRs) expressed on innate immune cells, including toll-like-receptors (TLRs) and NOD-like-receptors (NLRs) (3–5) although there are a breadth of potential innate activation mechanisms (1, 3–6). This capacity to enhance responses not only increases efficacy but can reduce the required quantity of antigen in vaccine formulations, enhancing supply in the case of pandemic infections (3). Thus, understanding how adjuvants modulate the immune response is key to providing a mechanism-based approach to rationally tailor vaccines. While non-adjuvanted vaccines are *usually* capable of inducing sufficient antibody responses, it is widely characterized that adjuvants are capable of enhancing and altering the quality of humoral responses (7). Furthermore, for some diseases, including malaria, HIV, and TB, antibody responses alone are not considered to be sufficient to eliminate the pathogen (8). Thus, adjuvants which can generate both robust CD4+ T cell and strong neutralizing antibody responses are required (9).

AS01 is a liposome-based vaccine-adjuvant containing two immunostimulants: monophosphoryl lipid A (MPL) and QS-21 (Antigenics LLC, a wholly owned subsidiary of Agenus Inc., a Delaware, USA corporation). MPL is a TLR4 agonist, and QS-21 is a saponin, derived from the *Quillaja saponaria* soap bark tree. This formulation has been shown to enhance both antibody and T helper 1 (Th1) responses to antigens (8, 10). It is currently employed in two approved vaccines against malaria and herpes-zoster virus (9). AS01-adjuvanted vaccines have shown high efficacy results in herpes-zoster phase III trials, where two doses result in >90% efficacy against herpes-zoster, regardless of age, including in >70 year old patients (11, 12), showcasing an ability to overcome the age-related defect associated with vaccination. Furthermore, in a phase 2b clinical trial, AS01-adjuvanted vaccines provided 54% protection against active TB (13). TLR agonists like MPL are often utilized in modern adjuvants, as they modulate the type and duration of the immune response (14–17). However, adjuvants can be greatly improved by the inclusion of additional immunostimulants, as observed with AS01, where, QS-21 is found to synergise with MPL. While MPL's mode-of-action (MoA) is widely characterized, QS21's MoA is not well-understood, although it has been shown to co-localize with subcapsular-sinus macrophages (SCS-M) leading to inflammasome activation in a caspase-1 dependent manner (18–21). Caspase-1 activation can trigger pyroptosis, activation of damage-associated-molecular patterns (22) and cleave pro-IL-1β and pro-IL-18 into bioactive pro-inflammatory IL-1β and IL-18 (23, 24). IL-1β is pleiotropic in function, rather IL-18 has a specific role in innate IFNγ production (25). AS01-adjuvanted vaccines induce an inflammatory response associated with transient production of innate IFNγ in the draining lymph nodes of mice, peaking at approximately 6 h post-injection (PI), and subsiding to baseline levels by 48 h PI (8). It has been shown that this early IFNγ production is required to promote a functional CD4+ T-cell response in mouse models (10). Furthermore, in humans, serum IFNγ is associated with clinical protection (22). However, some key questions remain regarding the MoA of AS01, such as the role of key early events in the adaptive response.

Genetically modified animal models have provided vital insight into the mechanistic processes underpinning vaccine efficacy. Over the past two decades, these models have been utilized to determine AS01 MoA. These pre-clinical models have permitted investigation of individual genes and proteins, in a reductionist manner (26), however not all mechanistic questions can be addressed in this way. Systems biology methodologies including machine learning, statistical, mathematical, and agent-based models can provide a holistic perspective on MoAs through data and knowledge integration (27–30). This can permit exploration of the relationships between different components in the biological system through simulation, where systems are not viewed purely as a sum of parts, but where additional phenomena can emerge as a result of integration. These methods can capture the complexity of the biological system allowing exploration of individual or population dynamics, the role of localized microenvironments, vaccination dose and time (31), and can be used to guide and optimize experimental design (27, 30, 32–36). This permits exploring dose modulation, prioritizing research avenues and determining experimental endpoints that maximize the value of individual animal experiments. Different systems-based approaches are increasingly being applied to biomedical research problems; permitting development of novel mechanistic hypotheses, spatio-temporal analysis of function of cytokines, chemokines, growth-factors, and cell-cell interactions that currently cannot be achieved *in vivo* (34–38).

Yet if systems-based modeling approaches are to add value to our understanding of the biological system, it is critical that the relationship between the biological understanding and how this knowledge is captured *in-silico* is understood. In the realms of immunology, our previous work has shown the adoption of a principled approach to the development of such tools, focusing on developing confidence that the model is fit for its purpose as providing a platform for exploring and contributing to our understanding of real-world biological systems (30, 35, 39–43).

**Figure 1:**
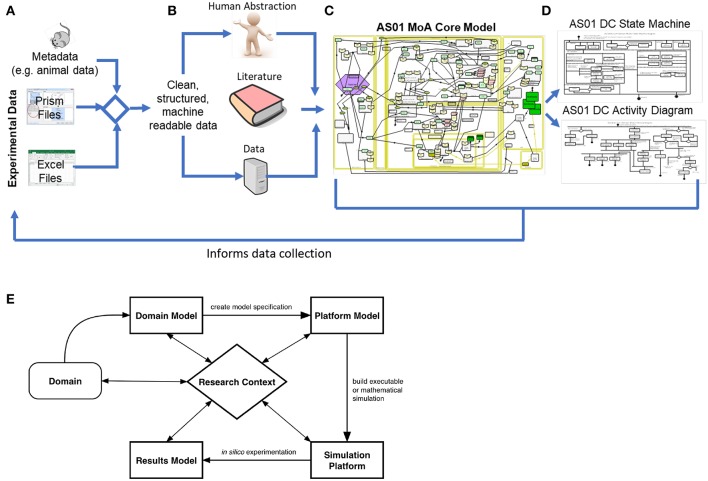
Development of the Domain Model: in this context, development of the domain model began with **(A)** the collection of experimental data from Graphpad Prism and Excel files, and the corresponding experiment metadata. Following this, an automated pipeline was developed to clean, structure, and integrate the experimental results and the corresponding metadata, resulting in machine-readable data. Next, human abstraction, the AS01 data and immunological literature were appropriately integrated **(B)**. The AS01 data informed the development of the model 2-fold, firstly by constituting the available evidence behind the captured processes, but also by informing the rationale underpinning any human abstraction required to inform gaps in understanding. The core MoA model **(C)** was utilized to scope the requirements of the lower-level models describing component function, and so from the core MoA model, state-machine and activity diagrams were developed **(D)**. Furthermore, Domain Model development can inform data collection by permitting the understanding of what data is required to ask the scientific question. **(E)** An overview of the CoSMoS process. The system of interest, in this context a biological system, is known as the domain. From this, the current scientific understanding of the system is modeled with respect to the research context, and encapsulated in the “Domain Model”. From this, and often after a process of refinement driven by the scientific question, a software blueprint called a Platform Model is developed. At this stage, further abstractions may be necessitated, based on available computational resources or model simplifications, which are decisions that should be documented as part of the Platform Model. Following this rigorous process builds confidence in the model and simulation, improving the likelihood that the simulation is a “fit for purpose” representation of the biological system (43–45). The simulation platform is an executable piece of software written in computer code, that implements the Platform Model, while the results model encapsulates the understanding that is generated from the simulation.

These concepts, however, have rarely been applied to exploring adjuvant MoAs, which are highly complex systems, spanning multiple organs, and levels of biological hierarchy. Thus, we present a framework in which we follow a principled modeling process (44) and collate knowledge surrounding AS01 MoA, which will then be used to construct simulations to explore key mechanisms of interest. We have captured our current consensus of AS01 MoA (see Figure 1) through interdisciplinary teamwork detailing the functionality of components underpinning how AS01 works, with focus on how the production of innate-IFNγ drives an adaptive response. This work, where a system of interest is identified, modeled, and scientific questions are elucidated, collectively comprises the “Discovery Phase” of the modeling process (44). The result is a “Domain Model” which is a model (i.e., an abstraction) of the key biological detail (44), which serves as a biological basis for simulation construction (Figure 1E). Domain models describe only the relevant biology, and do not describe concepts related to simulation construction, or how computer code is developed (44). Decisions on which modeling methods are utilized are taken subsequently during development of a separate model, named a “Platform Model,” where mathematical and computational concepts are introduced, detailing how the Domain Model is to be implemented as a simulation (44). In this perspective, we focus on the presentation of a framework for creating a non-executable Domain Model that captures and brings together understanding of AS01 biology, and present specific exemplars describing key component functions. To embrace simplicity, focus was placed on capturing essential components and entities where ample evidence of involvement in AS01 MoA is available. We believe that the application of this framework will complement work to explore AS01 MoA, and that these concepts should be utilized more generally to further understand adjuvant MoAs and thus enhance vaccine efficacy.

## Capturing the High-Level Consensus of AS01 MoA in a CellDesigner Model

Based on murine experimental data and a consensus understanding of AS01 knowledge we have developed a hypothesis of AS01's MoA. This was initiated with a focus on key questions in AS01 biology: “*In preclinical models, after intramuscular injection of a vaccine adjuvanted with AS01, how does AS01 initiate an immune response? What are the key interactions that give rise to the generation of an antigen-specific CD4*+ *T helper cell responses and subsequent antibody production? How does IFN*γ *regulate these different processes?*” To address these questions, we identified and captured key cells and processes in the model. The highly visual characteristics of the Systems Biology Graphical Notation (SBGN) (46) applied in CellDesigner (47) (www.celldesigner.org) allows for improved communications which is crucial when working in cross-disciplinary teams; permitting transparency, knowledge retention, and future reusability through linking AS01 data and wider immunological literature via diagrams of biological processes. The development process, which is described below, evolved a joint understanding between research teams, resulting in one shared model (Figure S1). This process permitted the confirmation of knowledge gaps, and the resulting consensus of AS01 MoA will inform the remaining development of the Domain Model. The following four step process (Figure 1A–C) was used to capture AS01 MoA in a process diagram: **(1)** Development of a biological “cartoon” incorporating current knowledge of how the adjuvant generates an adaptive immune response in a specific host; **(2)** The “cartoon” was used to generate a formal CellDesigner model; **(3)** An iterative process of CellDesigner model development was followed, to incorporate key team ideas, capturing, refining, and extending key aspects of the biology; **(4)** The final model was scrutinized, based on team discussions, resulting in a collective understanding agreed by all parties, to generate a single combined model of the adjuvant's MoA.

## A Description of the AS01 CellDesigner Model

While the CellDesigner model does describe some detail (these differences are depicted in yellow) of the response after a booster dose of an AS01-adjuvanted vaccine, it's main focus is the primary response. Thus, the following section describes the generation of a primary murine response to an antigen adjuvanted with AS01. As observed *in vivo*, in the CellDesigner model, after intramuscular injection of an AS01-adjuvanted-vaccine (Figure S1A) the adjuvant components both activate local cells and drain into the dLN (Figures S1C–E) initiating the immune response (8). In the muscle (Figure S1A), the model captures the abstracted hypothesis that MPL and QS-21 activate a ‘muscle-resident immune cell', which through chemokine secretion, recruits CCR2^+^ LY6C^hi^ Monocytes from the blood, into the injection site. These monocytes are capable of activation induced by MPL (48), antigen capture and migration into the dLN. This mechanism has been observed using lymphatic cannulation in an ovine (sheep) vaccination model with an AS01-adjuvanted vaccine (49). The potential for Monocytes to infiltrate via HEVs is not captured. Muscle-resident DCs (Figure S1A) are also capable of activation by MPL, capturing antigen, and undergoing a maturation process. To capture an abstraction of DC maturation, the key stages are distinguished in the model by an “immature DC”, a “maturing DC,” and a “mature DC.” Immature DCs in the model express TLR4 and IFNγ receptor. Maturing DCs also express CCR7 (50) which mediates migration from tissue to the LN paracortex (51), co-stimulatory molecules (CD80/86), IL-12 receptor and vaccine peptide antigen-MHCII (pMHC) complexes. In addition to the migration of DCs and monocytes into the LN, adjuvant and antigen free-flow from the injection site through the afferent lymphatics (AL) (Figure S1B) into the dLN (8). After arrival in the dLN, QS-21 co-localizes with CD169^+^ SCS-M, (Figure S1D) and can induce IL-18 secretion from these cells (18). In the paracortex (Figure S1C), secreted IL-18 delivers an activation signal to “innate IFNγ secretor cells” (ISC) (10, 25). This cell type is an abstraction to promote simplicity, encompassing Natural Killer cells, Natural Killer T-cells, innate-like CD8^+^ T-cells, ILC1s, and gamma-delta T-cells, which have all been shown to contribute to the early, innate production of IFNγ after AS01 stimulation (10). IL-18 stimulation of ISCs is capable of promoting the production of IFNγ, augmented by IL-12 (10). During a secondary response, IFNγ levels are further augmented by IL-2 derived from antigen-specific CD4+ T cells, further promoting synergistic production of IFNγ. Furthermore, SCS-M (along with follicular dendritic cells) can capture free-flowing antigen, and transfer it to B-cells in the follicle (52), contributing to their priming. The capacity of activated monocytes to differentiate into DCs (8). IL-12 in the model is hypothesized to be secreted by a pool of DCs, [including monocyte-derived-dendritic-cells (MoDCs)] and activated monocytes. At early time points, consistent with literature, IFNγ and IL-12 production is thought to promote differentiation of naïve cognate CD4+ T-cells toward a T Helper 1 (Th1) polarization (53). T helper cells are captured in the model, although there is an abstraction at this level for diagrammatic simplicity—the cell is a single entity, where no distinction is made between Th subsets. In the lower-level models, the appropriate distinctions between phenotype of these cells are captured. Here, the Th cell provides expansion, immunoglobulin switching and survival signal to B-cells, or secretes Th1-associated cytokines (TNF-α, IL-2, IFNγ). The model also captures the key stages of antigen-specific B cell priming, activation, and differentiation into “antibody-secreting-cells” or memory B cells, and the formation of germinal centers (Figure S1E). The blood compartment of the model (Figure S1F) captures the circulation of Th subsets after lymph node egress, and antibody circulation. The remit of the modeling exercise only requires the capture of the generation of T cell and antibody responses, and not the quality or functionality of the antibody response, nor the characteristics of the T-cell response in peripheral tissues, so these are not modeled (for inclusion and exclusion criteria, see Datasheet 2). Other inflammatory cytokines such as IL6, IL-1β etc. are produced during a response to AS01 however it is unclear how they contribute to early immune response to AS01.

## An Exemplar Capturing and Describing Key Components of AS01 MoA

**Figure 2:**
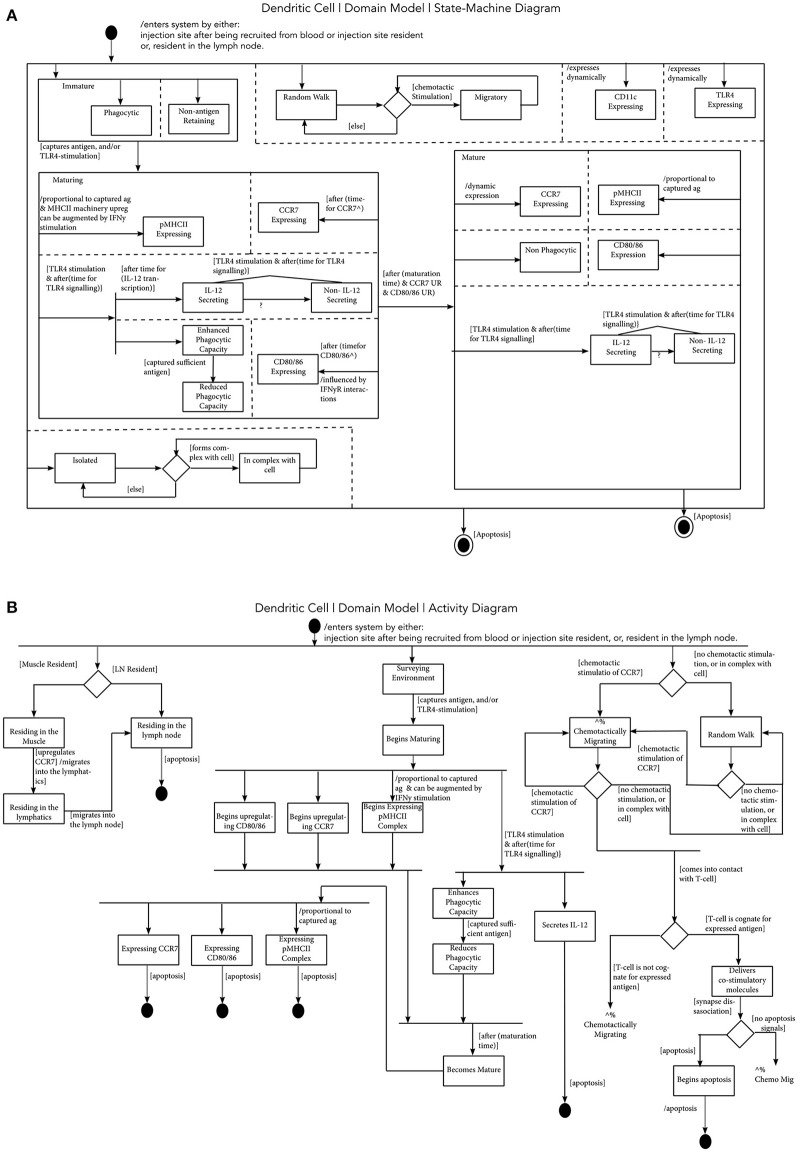
Modeling Dendritic Cell Function. **(A)** A Dendritic Cell State-Machine Diagram: states are represented by rectangular boxes, which may encompass smaller rectangular boxes, indicating parent and sub-states, respectively. A black circle indicates the initial state of the object, and the “double” circle indicates the final state. Arrows indicate transitions between the states, and information surrounded by square brackets describe a condition that must be satisfied for the transition to proceed. An expression preceded by a forward slash, indicates further information with respect to a state transition or a state. Finally, dashed lines represent concurrent states and a diamond indicates a decision is to be made, determined by a condition being satisfied. **(B)** A Dendritic Cell Activity Diagram: in this diagram, a black circle indicates the start of processes, and a double circle indicates the end of activities. The rectangles indicate activities and diamonds represent decisions. Horizontal black lines, are forks and joins, which indicate, respectively, the start and end of activities that occur concurrently.

To construct a simulation, lower-level behaviors, function, and interactions of components (cells and cytokines) must also be captured, thus substantiating the model. With respect to the research questions, we aim to capture an appropriately detailed description of the biology, building on, and informed by the scope of the CellDesigner model. An adapted version of the unified modeling language (UML) was employed to develop “state-machine” and “activity” diagrams (Figures 2A,B) (34, 35). Thus, the Domain Model comprises the CellDesigner visualization and state and activity diagrams. State-machine diagrams describe the different states a component (entity) can exist in, and requirements that govern transitions between these states. Activity diagrams in this context describe activities and events in the system that emerge from interactions between cellular components. Care must be taken to capture the relevant biology, and where abstractions are made, these should be appropriate, and decisions behind inclusion and exclusion of functionality and rationale for abstractions should be clearly documented to maintain a trail of the considerations involved, providing transparency of the decisions behind model development in order to permit scrutiny. Our laboratories have developed a tool for this purpose and the reader is directed to Alden et al. (42) for a detailed explanation.

Figure 2A is a state-machine diagram for a murine DC. This captures an abstraction of a cell type, encompassing the different DC subsets in the model (including MoDCs). This diagram captures the key functionality, at the required level of complexity of the DC for our research context. This begins with the cell entering the system, resident in either the injection site or in the dLN. Initially, the cell is immature, and whilst immature, it is phagocytic, and doesn't retain antigen (54). If it captures antigen and/or is stimulated by a TLR4 agonist, it enters the “maturing” state, where the DC has varying phagocytic capacity dependent on levels of antigen it has acquired, increases expression of pMHCII complexes, CCR7, CD80/86, and secretes IL-12. The increased expression of CCR7 allows the cell to chemotactically migrate toward CCL19/21 gradients in both draining lymphatics and in the dLN paracortex. After the time required to undergo full maturation, and if there is sufficient upregulation of CCR7 and CD80/86, the cell can become fully mature. When a DC is fully mature, it is not phagocytic, expresses high levels of pMHCII complexes, CCR7, CD80/86, and can secrete IL-12. During all life stages, this cell dynamically expresses CD11c, and TLR4. Mature DCs can also present antigen to T cells, as the diagram also captures the ability for a DC to be isolated or in a complex with another cell (i.e., when presenting antigen), and to be undergoing random or chemotactic migration. The cell exits the system if it undergoes apoptosis.

Activity diagrams are developed to describe actions and interactions of cellular components. In Figure 2B an activity diagram is shown for a murine DC. Initially, the cell is either muscle or dLN resident, surveying the environment, and either undergoing random or chemotactic migration. Following exposure to antigen, and/or stimulation by a TLR-4 agonist, the DC can begin maturing, as described in the state-diagrams, and can eventually become fully mature. If a cell is resident in the muscle site, due to the upregulation of CCR7, it can begin migration into the lymphatics, toward the dLN. If the cell is resident in the dLN, the upregulation of CCR7 would direct it toward the CCL19/21 gradients (which functionally, would direct it to the T cell area) (50). If the DC comes into contact with a T cell, and it undergoes a cognate interaction, the cell can deliver co-stimulatory molecules promoting activation, and either undergo apoptosis or return to migration. Not shown in this perspective, this same approach has been applied to all cell types and processes captured in the AS01 MoA CellDesigner model (see Datasheet 1 for a list of diagrams).

## Modeling and Simulation as a Basis for Exploring Adjuvant MoA

Following the process outlined in this perspective, simulation can be utilized to integrate knowledge and explore hypotheses underpinning biological systems. The development of Domain Model diagrams can undergo iterative refinement driven by specific scientific questions, resulting in a domain model appropriate to address a specific question. Following this, a Platform Model is developed, and subsequently, a simulation can be constructed written in computer code (44). Simulations are then calibrated to real-world data, and usually undergo a process of validation. After construction, simulations can be inspected by a variety of analysis techniques, such as sensitivity and robustness analysis, permitting an exploration of the effects of stipulated immunological behaviors on the system (41, 45). This can elucidate important MoAs, which can be explored and validated *in vivo* (29, 35), thus guiding experimental design. Furthermore, systems-based techniques that explore optimization could be used to elucidate more efficient dosing schedules. For detailed reading on the entire modeling process described here, the reader is directed to Andrews et al. (44).

## Conclusion

We have presented a framework to capture and collate MoA knowledge and applied it to integrating and exploring AS01 MoA. This framework has informed the development of a Domain Model, capturing high-level AS01 MoA using CellDesigner, which was further substantiated through UML diagrams describing lower-level functionality. The CellDesigner visualization is an explicit description of the key, higher-level biology, resulting in a visualization with which researchers can illustrate and share their ideas and communicate knowledge and knowledge gaps. Building upon this, the UML-like diagrams captures detailed knowledge and hypotheses underpinning the system, bringing together AS01 understanding, immunological literature, and rational assumption to describe lower-level component behaviors. The resulting Domain Model not only brings together understanding about the biological system, but after appropriate refinement driven by a specific scientific question, can serve as a biological basis to construct simulations, permitting exploration of key research questions. We believe that these concepts will complement work on AS01 MoA and envision that these 3Rs-based approaches (https://www.nc3rs.org.uk/the-3rs), through viewing data holistically and complementing *in vivo* experimentation, can be applied more generally to improve the understanding of other adjuvant MoA, thus enhancing vaccine efficacy.

## Author Contributions

PB, JT, KA, CC, AC, CA, MCC, AD, RM, and MC were involved in the conception and design of the study. CC, AC, CA, and MC acquired the data. CC, AC, CA, MCC, PB, JT, and KA analyzed the data. PB, AD, RM, ST, MC, JT, KA, CA, and MCC analyzed and interpreted the results. All authors were involved in drafting the manuscript or critically revising it for important intellectual content. All authors had full access to the data and approved the manuscript before it was submitted by the corresponding authors.

### Conflict of Interest Statement

CC, AC, CA, RM, AD, ST, and MC are employees of the GSK group of companies. CA, RM, AD, and ST report ownership of GSK shares and/or restricted GSK shares. MC was supported by a Marie Sklowdoska Curie Intra-European Fellowship (ref. “ADJSYN”). PB was holding a Ph.D. studentship and collaborated with GSK at the time of the study as part of his Ph.D. training. PB's work was partially funded by GlaxoSmithKline Biologicals SA through a post-graduate studentship. The remaining authors declare that the research was conducted in the absence of any commercial or financial relationships that could be construed as a potential conflict of interest.

## Supplementary Material

Figure S1 The CellDesigner Model: an interactive model is available in Datasheet 1 (*requires CellDesigner installation*). This model captures the following key compartments: the muscle injection site **(A)**, the lymphatic vessels **(B)**, the draining lymph node (dLN) [including paracortex **(C)**, B cell follicles **(D)**, germinal center **(E)**], the blood **(F)**, and the bone marrow **(G)**. This model captures high-level mechanisms that are hypothesized to give rise to the phenomena observed by experimentation in mice. This begins with the intramuscular injection (34) of the vaccine at time zero **(A)** and captures the events leading to the secretion and circulation of antibodies **(E,F)**, and lymph node egression of effector memory T cells (9).

Datasheet 1 Full list of Domain Model Diagrams: the full list of diagrams that were created for the AS01 MoA Domain Model.

Datasheet 2 A list of the entire Core AS01 MoA Domain Model Diagrams.
