# Erratum: Application of Modeling Approaches to Explore Vaccine Adjuvant Mode-of-Action

**DOI:** 10.3389/fimmu.2019.02905

**Published:** 2019-12-02

**Authors:** Frontiers Production Office

**Affiliations:** Frontiers Media SA, Lausanne, Switzerland

**Keywords:** vaccines, adjuvants, mathematical modeling, computational biology, systems biology, mechanistic modeling, AS01

Due to a production error, the author “Margherita Coccia” was not added in the **Conflict of Interest Statement**. The correct statement should read:

“CA, RM, AD, MC, and ST report ownership of GSK shares and/or restricted GSK shares.”

Furthermore, there is a typo in the city of Affiliation 4. Instead of “Faculty of Technology, University of Sunderland, Sunderlandm, United Kingdom” it should be “Faculty of Technology, University of Sunderland, Sunderland, United Kingdom.”

Lastly, the caption provided in **Figure S1** is incorrect. The correct caption should read:

“**Figure S1**. The CellDesigner Model captures the following key compartments: the muscle injection site **(A)**, the lymphatic vessels **(B)**, the draining lymph node (dLN) [including paracortex **(C)**, B cell follicles **(D)**, germinal center **(E)**], the blood **(F)**, and the bone marrow **(G)**. This model captures high-level mechanisms that are hypothesized to give rise to the phenomena observed by experimentation in mice. This begins with the intramuscular injection (34) of the vaccine at time zero **(A)** and captures the events leading to the secretion and circulation of antibodies **(E,F)**, and lymph node egression of effector memory T cells (9).”

The publisher apologizes for these mistakes. The original article has been updated.

