# Supplementary figures and images for: Application of Modeling Approaches to Explore Vaccine Adjuvant Mode-of-Action
Source: Front Immunol. 2019 Sep 12;10:2150. doi: 10.3389/fimmu.2019.02150 (PMC6751289; doi:10.3389/fimmu.2019.02150)

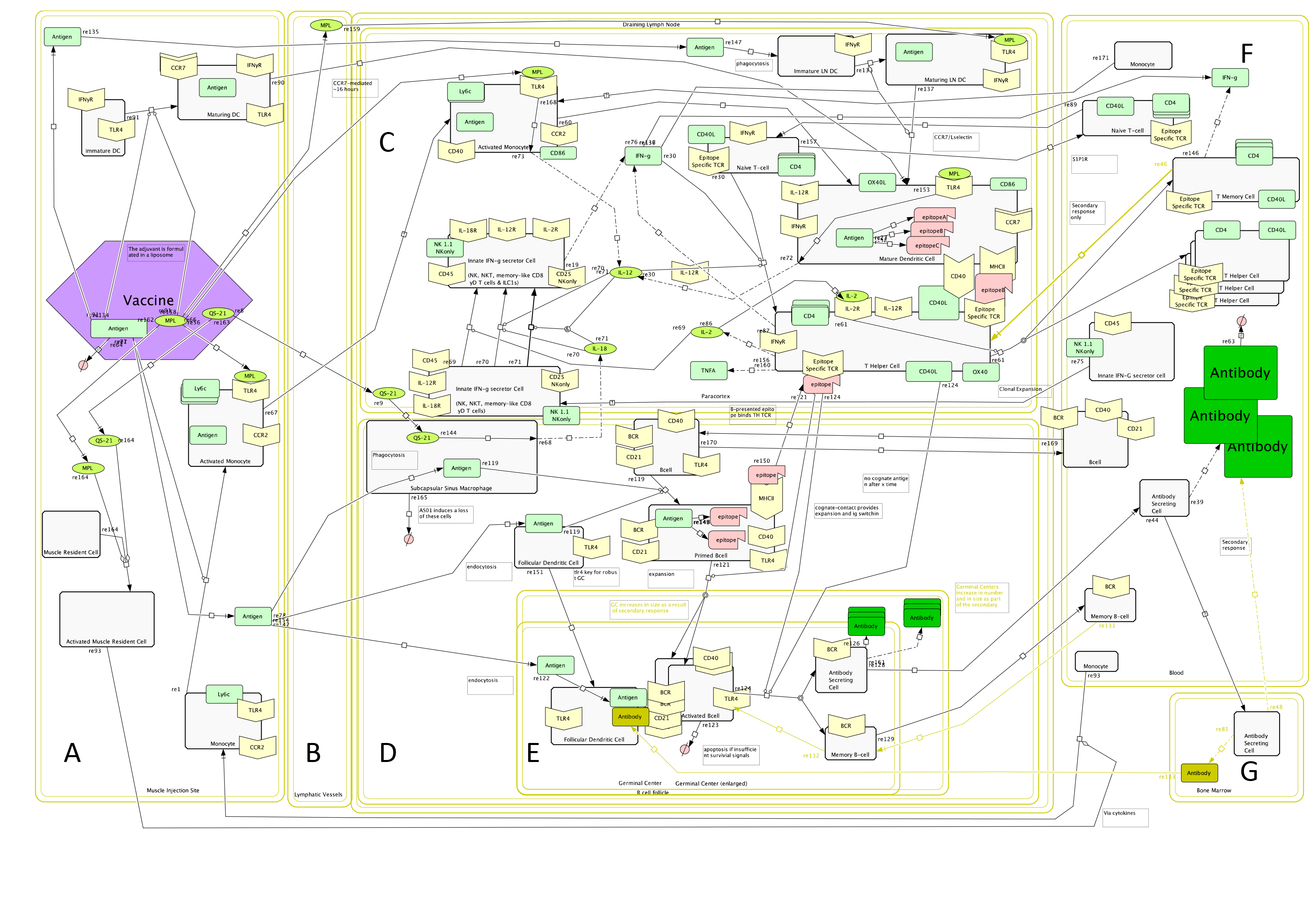

Supplement: Figure S1 — The CellDesigner Model: an interactive model is available in Datasheet 1 (requires CellDesigner installation). This model captures the following key compartments: the muscle injection site (A), the lymphatic vessels (B), the draining lymph node (dLN) [including paracortex (C), B cell follicles (D), germinal center (E)], the blood (F), and the bone marrow (G). This model captures high-level mechanisms that are hypothesized to give rise to the phenomena observed by experimentation in mice. This begins with the intramuscular injection (34) of the vaccine at time zero (A) and captures the events leading to the secretion and circulation of antibodies (E,F), and lymph node egression of effector memory T cells (9). [file Image_1.TIF]
