# Supplementary material for: Application of Modeling Approaches to Explore Vaccine Adjuvant Mode-of-Action
Source: Front Immunol. 2019 Sep 12;10:2150. doi: 10.3389/fimmu.2019.02150 (PMC6751289; doi:10.3389/fimmu.2019.02150)
Supplement: Datasheet 1 — Full list of Domain Model Diagrams: the full list of diagrams that were created for the AS01 MoA Domain Model. [file Data_Sheet_1.PDF]

Domain Model Diagrams: Full list

- Expected behaviours diagram:
    - Describing the research context, the phenomena observed in the system by experimentation, and the behaviour that is expected to cause the observable phenomena to manifest. This is then supported by a holistic hypothesis.
- State machine and activity diagrams for the following cell types:
    - Muscle-resident cell (abstraction for Muscle resident macrophages, skeletal muscle cells, epithelial cells etc, which secrete factors initiating Monocyte and Neutrophil extravasation from the blood).
    - Monocyte
    - Dendritic Cell
    - CD169+ F4/80- Subcapsular Sinus Macrophage
    - IFNγ producer cell (comprising Natural Killer cells, Natural Killer T-cells, innate-like $CD8^+$ T-cells, ILC1s and gamma-delta T-cells)
    - Naïve CD4+ T cell
    - T Helper Cell
    - Follicular Dendritic Cell
    - B cell
    - Antibody-secreting-cell
    - Memory B Cell
    - T Memory Cell
- Additional activity diagram for:
    - IFNg
    - Integrated injection Site dynamics
    - Integrated Lymph node dynamics
