# Supplementary material for: Application of Modeling Approaches to Explore Vaccine Adjuvant Mode-of-Action
Source: Front Immunol. 2019 Sep 12;10:2150. doi: 10.3389/fimmu.2019.02150 (PMC6751289; doi:10.3389/fimmu.2019.02150)
Supplement: Datasheet 2 — A list of the entire Core AS01 MoA Domain Model Diagrams. [file Data_Sheet_2.PDF]

| Entity | Included or Excluded | Justification | Further Notes and Relevant References |
| --- | --- | --- | --- |
| Muscle-resident Cell | Included | It is hypothesised that cells resident in the muscle (potentially skeletal muscle, Macrophages or Endothelial cells) are activated by AS01 upon injection, resulting in the production of chemokines that recruit Neutrophils and Monocytes. This entity is included to capture this at a high-level. | |
| CD169+ F4/80- Macrophages | Included | Subcapsular sinus Macrophages have been shown to release IL18 after QS21 mediated activation of caspase-1. IL18 contributes to the production of IFNγ by NK cells. In addition, this cell can capture antigen and transfer it into the B cell follicles, although this has not been tested in the context of AS01 vaccination. | Detienne 2016 Sci Reports<br>Coccia, NPJ Vaccine 2017 |
| IFNg Secretor Cell | Included | This is an abstraction that captures various cell types shown to secrete IFNγ at early time points after AS01 injection (see Figure S2). Inclusion of this entity is vital as IFNg has been shown to promote CD4+ T cell responses to AS01. | Coccia, NPJ Vaccine 2017. |
| Monocytes | Included | In a sheep cannulation model, it has been demonstrated that after intramuscular injection of AS01, Monocytes infiltrate the dLN in large numbers. Their function in this context is not yet clear, however we hypothesise that they secrete IL12, and capture their ability to differentiate into DCs. | MoDCs detected in Didierlaurent, JI 2014.<br><br>Sheep cannulation vaccination model in Neeland, JI, 2016.<br><br>IL12 production evidence (external to AS01) in De Koker, Scientific Reports, 2017. |
| Dendritic Cells | Included | The enhancement of adaptive immunity by AS01 has been shown to depend on Activated Dendritic Cells. | Didierlaurent, JI 2014 |
| IFNγ | Included | IFNγ has been shown to be a significant component of the response to AS01. Whilst questions remain on its function in this context, it’s transient production has been shown to be critical for the generation of cytokine-producing CD4+ T cells after AS01-based vaccination. | Coccia, NPJ Vaccine 2017.<br><br>Additionally, neither of the AS01-components (MPL nor QS21) alone induce early IFNγ production (Coccia, NPJ Vaccine 2017) |

| | | | |
|---|---|---|---|
| IL12 | Included | IL12 has been shown to synergise with IL18 to promote optimal production of IFNγ after vaccination with AS01. | Coccia, NPJ Vaccine 2017. |
| IL18 | Included | IL18 has been shown to synergise with IL12 to promote optimal production of IFNγ after vaccination with AS01. | Coccia, NPJ Vaccine 2017. |
| T Helper Cell | Included | CD4+ T cells are induced upon AS01 vaccination. | Coccia, NPJ Vaccine 2017. |
| Follicular Dendritic Cells | Included | While their role has not been elucidated through experiments with AS01, fDCs are a key component of the adaptive response. They secrete CXCL13 which organises B cells in the follicles and attracts CXCR5+ T Helper cells towards the B / T border. Further, fDCs can capture and express antigen. | |
| B cell | Included | There are scant data available on B cell responses to AS01, however as the synthesis of antibody is captured as it is a critical component of vaccination. Thus canonical, high-level B cell dynamics are captured to facilitate the synthesis of antibody. | |
| Antibody-Secreting-Cell | Included | ASCs are included as a key component of antibody synthesis. | |
| Memory B Cell | Included | Memory B cells are included to capture basic dynamics of the secondary immune response. | |
| Memory T Helper Cell | Included | Memory T Helper cells are included to capture basic dynamics of the secondary immune response. | |
| IL6 | Excluded | IL6 is a pleiotropic cytokine with both anti-inflammatory and pro-inflammatory functions. While IL6 is produced after AS01 vaccination in mice, it’s role is unclear and thus not included at this stage. | |
| Neutrophils | Excluded | There is no evidence that Neutrophils contribute to the IFNγ production or IFNγ modulation of the adaptive response to AS01. | Furthermore, there are conflicting data on the role of Neutrophils during the immune response in lymph nodes. |
| CD4+ T Helper 17 | Excluded | There is no evidence that IL17-producing CD4+ T cells contribute to the production of IFNγ or modulation of the adaptive response to AS01. | IL17-producing CD4+ T cells are observed upon M27/AS01E vaccination (Penn-Nicholson, Vaccine 2015). |

| | | | |
|---|---|---|---|
| Regulatory CD4+ T cells | Excluded | Even if these cells are induced by AS01, we are unsure of their contribution to the research context and the mechanism by which they function. | |
| Antigen-Complement complexes | Excluded | Antigen-C3dg complexes are critical for driving the high affinity antibody response through binding to of C3dg-antigen complexes to CD21 on FDCs, however because the model is not addressing mechanistic questions on the germinal centre response, which is C3dg dependent, complement has not been included in the model. | |
